# Complex temporal dynamics of mental health indicators: A longitudinal network approach perspective

**DOI:** 10.1017/S0033291725102080

**Published:** 2025-10-03

**Authors:** Matúš Adamkovič, Benjamin Simsa, Bibiána Jozefiaková, Gabriela Mikulášková, Peter Babinčák, Gabriel Baník, Jaroslava Bočanová, Denisa Fedáková, Klára Kačmariková, Pavol Kačmár, Michal Kentoš, Viktória Majdáková, Lenka Vargová, Ľubica Zibrínová, Ivan Ropovik

**Affiliations:** 1 Centre of Social and Psychological Sciences, Slovak Academy of Sciences, Bratislava, Slovakia; 2 Faculty of Humanities and Social Sciences, University of Jyväskylä, Jyväskylä, Finland; 3 Faculty of Education, Charles University, Prague, Czechia; 4 Faculty of Arts, Pavol Jozef Šafárik University in Košice, Košice, Slovakia; 5 OUSHI, Palacky University, Olomouc, Czechia; 6 Faculty of Arts, University of Presov, Prešov, Slovakia; 7 Faculty of Social Studies, Akademia Humanitas, Sosnowiec, Poland; 8 Department of Psychology and Social Sciences, Ambis University, Prague, Czechia; 9 Czech Academy of Sciences, Institute of Psychology, Prague, Czechia

**Keywords:** anxiety, depression, insomnia, longitudinal analysis, mental health, network analysis, panel networks, posttraumatic stress disorder

## Abstract

**Background:**

Although mental disorders have long been considered complex dynamic systems, our understanding of the mutual interactions and temporal patterns of their symptoms remains limited.

**Methods:**

In this longitudinal study, we examined the structure and dynamics of four key mental health indicators – depression, anxiety, post-traumatic stress disorder, and insomnia – in a representative sample of the Slovak population (effective *N* = 3,874) over 10 waves spanning 3.5 years. For each construct, a longitudinal panel network model was estimated.

**Results:**

The temporal relationships between symptoms were mostly weak, with the autoregressive effects typically being stronger. In depression, anxiety, and insomnia, some causal chains and feedback loops were identified. In all constructs, both contemporaneous and between-person networks showed dense connections.

**Conclusions:**

The findings provide critical insights into the complexity of mental health development, offering potential targets for intervention and prevention strategies.

## Introduction

Mental health is a critical determinant of overall health and well-being, impacting whole societies and even economies (The Lancet Global Health, 2020; WHO, 2022). Understanding its temporal dynamics is crucial for prevention, treatment, and general prediction of population health trends. The long tradition of applying a medical reductionist paradigm to mental disorders (i.e., the assumption of the existence of a hidden unitary cause of psychopathology), however, seems insufficient for further advancing the comprehension of psychopathology and the development of effective treatments (Adam, 2013). Recently, a mutualism framework – network theory – that describes mental disorders as complex systems arising from causal interactions among symptoms (e.g. Borsboom, 2008; Borsboom, 2017; Borsboom & Cramer, 2013) has been shown to represent the ontology of mental disorders in a more nuanced way. It helps identify, for instance, the core and peripheral symptoms of a disorder, how the symptoms are interconnected and clustered, the efficiency of treatment, or even the intra-individual dynamics of psychological processes. The network approach has thus become a prominent perspective for studying psychopathology, with its adoption rapidly growing. Among the empirical studies utilizing the network approach, the majority (about 80%) analyzed cross-sectional data (Robinaugh, Hoekstra, Toner, & Borsboom, 2020), followed by studies focused on intensive longitudinal data designs capturing trajectories of symptom development at the level of an individual (Wysocki, van Bork, Cramer, & Rhemtulla, 2022). However, more generalizable nomothetic inferences about psychopathology dynamics require collecting data from many people across several time points. The data from such designs can be analyzed using specific techniques, such as panel graphical vector autoregression models (panel GVAR; Epskamp, Hoekstra, Burger, & Waldorp, 2022) or cross-lagged panel network models (Wysocki et al., 2022). The scarcity of research utilizing panel networks might be explained by the lack of available analytic tools. Recently, such tools (Epskamp, 2023; Wysocki et al., 2022) have been developed, allowing researchers to fill in the gaps in the literature by integrating complexity, temporal aspects of psychopathology, and the capacity to handle data from large samples.

### Network approach

The typical network models of psychopathology are graphical representations of symptoms and their mutual connections. Symptoms are represented as nodes, while edges reflect the estimated connections between nodes. In cross-sectional networks, the connections are usually modeled as undirected regularized partial correlations. Longitudinal network models allow examining the directionality of the relationships over time, including autoregressive effects. For this study, panel GVAR models (Epskamp, Waldorp, Mõttus, & Borsboom, 2018; Epskamp et al., 2022) are utilized. Panel GVAR estimates three distinct symptom networks (e.g. Martončik, Ropovik, & Adamkovič, 2024): (1) A temporal network model captures directed temporal dependencies of the nodes; that is, how a node at time point *t* predicts another node or itself at time *t* + 1 (i.e., directional relationships over time), while controlling for all other associations at time *t.* (2) A contemporaneous network is formed by estimating residual (co)variances after accounting for the temporal (lagged) effects, thereby capturing the partialized relationships between nodes within the same time frame, independent of their prior influences (i.e., symptom co-occurrence within the same person at a given time). It is noteworthy that in panel GVAR, “contemporaneous” associations are residual covariances after controlling for specified lagged effects; very fast dynamics (faster than the chosen lag) may still inflate or alter these residuals (Epskamp et al., 2018). (3) A between-person network illustrates the connections among stationary averages across individuals, accounting for the mean levels of all other nodes within the network (i.e., stable associations between average symptom levels across individuals).

### Mental health indicators

The present study focuses on four major mental health indicators: depression, anxiety, post-traumatic stress disorder (PTSD), and insomnia. These conditions share several overlapping features, including disturbances of mood, cognition, and physiological functioning. For example, insomnia is a common symptom of depression, anxiety, and PTSD, and has been shown to predict the onset of depression (Blanken, Borsboom, Penninx, & Van Someren, 2020). Similarly, negative affect and cognitive disturbances, such as difficulty concentrating, are central to both depression and anxiety (Wichers, Riese, Hodges, Snippe, & Bos, 2021; Wu et al., 2023). The symptomatology and dynamics of these constructs are described below.

Depression is one of the most common mental disorders, with lifetime prevalence rates estimated to range from 5 to 21% globally (e.g. Hasin et al., 2018; Lim et al., 2018; WHO, 2023). The core symptoms of depression, as defined in the Diagnostic and Statistical Manual of Mental Disorders, Fifth Edition (DSM-5) and International Classification of Diseases, 11^th^ Revision (ICD-11), are persistently depressed mood and/or loss of interest or pleasure. The available empirical evidence on symptom centrality suggests that energy loss, anhedonia, persistent depressed mood, and concentration problems constitute the core symptoms of depression, with studies indicating dynamic associations between these symptoms over time (Boschloo, Schoevers, van Borkulo, Borsboom, & Oldehinkel, 2016; Wichers et al., 2021). According to a longitudinal network study by Savelieva, Komulainen, Elovainio, and Jokela (2021), sad or persistent depressed mood, diminished interest in activities, and suicidal ideation were the most central symptoms over time, with persistent depressed mood and anhedonia being the most reactive to changes in other symptoms. While stronger network connectivity was not consistently associated with higher symptom levels or future depressive symptoms, micro-level studies provided support for the connectivity hypothesis, highlighting the complex interplay of depressive symptoms (Wichers et al., 2021). Depression is frequently comorbid with anxiety (Kalin, 2020).

Affecting approximately 4% of the global population, anxiety disorders are among the most common mental health conditions worldwide (Javaid et al., 2023; Spitzer, Kroenke, Williams, & Löwe, 2006). Generalized anxiety disorder (GAD) is characterized mainly by excessive, free-floating worry about potential adverse events that cause behavioral, cognitive, or somatic disturbances. The developmental trajectories of anxiety disorders are highly heterogeneous (Nelemans et al., 2014), and these conditions tend to be chronic and recurrent (Schweizer, 1995). Surprisingly, little is known about the structure and dynamics of GAD symptoms as a standalone construct, as available studies often combine it with other comorbid disorders (typically depression; e.g. Bekhuis, Schoevers, van Borkulo, Rosmalen, & Boschloo, 2016; Curtiss, Ito, Takebayashi, & Hofmann, 2018; McElroy, Fearon, Belsky, Fonagy, & Patalay, 2018). In a study on at-risk medical staff, Wu et al. (2023) observed an average connection of .10 between the symptoms, with the inability to regulate worries playing the most central role within the disorder. In another study, Hoffart, Burger, Johnson, and Ebrahimi (2023) analyzed temporal networks of generalized anxiety in the context of the coronavirus disease 2019 (COVID-19) pandemic and found that anxiety, generalized worry, and uncontrollable worry are strongly interconnected in both temporal and contemporaneous networks. Similarly, temporal network analysis revealed positive lagged relationships and feedback loops among GAD symptoms, with sleep disturbance exhibiting the highest out-strength centrality (Peng et al., 2024). These findings highlight the critical role of sleep disturbance in the development and maintenance of GAD. The etiology of anxiety disorders is closely linked to external factors such as stressful events (Zinbarg, Williams, & Mineka, 2022). Prolonged or intense stress can be perceived as trauma, potentially leading to PTSD (Wilson & Keane, 2004).

PTSD can develop after exposure to a traumatic event, with global prevalence rates ranging between 3% and 8% (e.g. Koenen et al., 2017), depending on geographic location and specific characteristics of studied populations (e.g. Gielen, Havermans, Tekelenburg, & Jansen, 2012). PTSD manifestations includes symptom categories such as intrusions, avoidant behavior, negative cognitive and mood changes, and hyperarousal. Compared to the DSM-5, the ICD-11 narrows its focus to avoidance, re-experiencing, and a sense of ongoing threat. Recent network studies have identified psychological reactivity, intrusive traumatic memories, detachment, and disinterest in activities as core symptoms of PTSD (e.g. Fried et al., 2018). Conversely, amnesia consistently shows low severity and centrality across datasets and networks (Fried et al., 2018; Isvoranu, Epskamp, & Cheung, 2021). Freichel, Herzog, and Billings (2024) revealed a reinforcing cycle in PTSD in which intrusion symptoms strongly predict avoidance and heightened threat perception. Hyperarousal symptoms have been shown to predict other PTSD symptoms over time, while avoidance symptoms are influenced by other symptom clusters (Schlechter, Hellmann, McNally, & Morina, 2022). Similarly, hypervigilance has been identified as a key predictor of symptom changes over time, connecting with at least one symptom from each DSM-5 PTSD cluster (Stefanovic, Takano, Wittekind, & Ehring, 2024). PTSD is also frequently comorbid with disorders like major depressive disorder and GAD, leading to disturbances in sleep (Leskin, Woodward, Young, & Sheikh, 2002). The longitudinal study by Keane et al. (2014) provides evidence for the temporal stability of PTSD, which extends to its network structure (Crowe, Harper, Moshier, Keane, & Marx, 2023). These findings emphasize the importance of specific symptoms, such as intrusion and hyperarousal, in driving the development and maintenance of PTSD.

Sleep disturbances are a common element across all three of the previously discussed constructs (Leskin et al., 2002; Merrill & Gibbons, 2023). Among these, insomnia stands out as a particularly prevalent sleep disorder, affecting around 22% of the general population (Zeng et al., 2020). It is characterized by difficulty falling asleep, staying asleep, or experiencing poor sleep quality that impairs daytime functioning (WHO, 2023). Core symptoms include difficulty falling asleep, frequent nighttime awakenings, and associated symptoms such as dysphoria and irritability (Hu, Li, Huang, Yan, & Huang, 2020; Takano, Ibata, Nakano, & Sakano, 2023). However, much of the existing research focuses on specific populations, such as workers at risk for insomnia (Takano et al., 2023) or mental health professionals during the COVID-19 pandemic (Bai et al., 2021). Sleep problems often occur as concomitant symptoms of other psychiatric or somatic conditions. For instance, difficulty falling asleep has been shown to predict the first onset of depression (Blanken et al., 2020). Longitudinal analyses reveal the dynamic nature of insomnia, particularly during the transition from adolescence to adulthood, characterized by relatively high rates of both remission and incidence (Hysing et al., 2020). According to Morin et al. (2009), 46% of individuals continue to experience insomnia after 3 years, while 54% report remission.

### Present study

Mapping the dynamics in the population’s mental health is crucial. However, for a more accurate representation of these dynamics, the complexity of these processes should be considered. To account for this, we utilize a longitudinal network approach to concurrently study both the complexity and dynamics of depression, anxiety, PTSD, and insomnia in a representative sample of the Slovak population. We study these mental health constructs on the symptom level and examine how symptoms within a disorder mutually affect each other over time.

## Methods

### Participants and data collection

The study was part of a broader project focused on the (post)pandemic mental health of Slovak inhabitants (APVV-20-0319). The longitudinal design of the study encompassed 10 waves of data collection over the period of 3.5 years, from August 2020 to December 2023. A representative sample of Slovak inhabitants, based on quota characteristics for gender, age, region, and education, was recruited. The data were collected online by a specialized agency. Altogether, 4,345 unique subjects participated in the study, with a mean of 1,989 participants per data collection wave. To ensure data quality, the careless-responding screening was based on a combination of improbably fast response times, failed attention checks, multivariate outliers (Mahalanobis distance), and long sequences of identical responses (longstrings). Across the waves, about 5–11% of participants were excluded because of these patterns.

The cleaned data set contains 3,874 unique participants, which translates to, on average, 1,758 participants per wave, with a median between-wave overlap of 59.7% (median wave-to-wave overlap of 76%). We examined whether higher mental-health scores at time *t* predicted dropout at *t + 1* (and across other lags) and found small effects of mental health scores on dropout status: depression *g* = 0.11 (SD = 0.09), anxiety *g* = 0.13 (SD = 0.10), PTSD *g* = 0.00 (SD = 0.13), and insomnia *g* = 0.10 (SD = 0.10). Overall, about 54.2% of the participants were female, and < 1% identified as other/nonbinary. The mean age at the baseline was 40.3 years (SD = 14.8). More than two-thirds (67.3%) of the sample had a job during most of the data collection period. Two-thirds of the participants (66.6%) had a partner during most of their participation. For per-wave sample characteristics, between-waves overlap, demographic characteristics before and after excluding participants who exhibited careless responding patterns, and differences in mental health by dropout status, see Supplementary Tables 1A–1D. Further insights into the study’s design and sampling methodology can be accessed at https://osf.io/32rsx. A detailed timeline mapping the situation in Slovakia (e.g. details on the course of the pandemic, the situation related to the war in Ukraine, etc.) is available at https://osf.io/vjmfd.

Participants were compensated for their time with rewards from the agency’s internal reward system and an extra lottery. Ethics approval was granted by the ethics committee at the Centre of Social and Psychological Sciences, Slovak Academy of Sciences.

### Measures

To minimize common method variance bias, both the order of the mental health scales and the order of the items within each scale were randomized. Depression was measured using the Quick Inventory of Depressive Symptomatology (QIDS-SR-16; Reilly, MacGillivray, Reid, & Cameron, 2015; Rush et al., 2003), which contains 16 items assessing 9 symptoms (a 4-point response scale indicating symptom severity) based on DSM-IV criteria, with internal consistency ranging from *ω* = .77 to .86 across the waves. Anxiety was assessed with the General Anxiety Disorder-7 (GAD-7; Löwe et al., 2008; Spitzer et al., 2006), a 7-item scale that rates symptom frequency (a 4-point response scale). The reliability of the scale ranged from *ω* = .89 to .93. To identify participants who were exposed to a traumatic event, trauma screening using a slightly modified Stressful Life Events Screening Questionnaire (SLESQ; Elhai et al., 2012; Goodman, Corcoran, Turner, Yuan, & Green, 1998) was employed. Participants who indicated experiencing a traumatic event were subsequently administered the PTSD Checklist for DSM-5 (PCL-5; Blevins, Weathers, Davis, Witte, & Domino, 2015; Forkus et al., 2023; Weathers et al., 2013), a 20-item measure assessing PTSD symptoms and their severity (a 5-point response scale), with reliability ranging from *ω* = .95 to .97. The trauma screening and PTSD assessment were done in 7 out of 10 waves. The PTSD networks were estimated on a subset of participants who indicated experiencing a traumatic event at the fourth measurement occasion (57.6%), maximizing both sample size and cross-wave participation. Insomnia was assessed using the 7-item Insomnia Severity Index (ISI; see Manzar, Jahrami, & Bahammam, 2021; Morin, Belleville, Bélanger, & Ivers, 2011), assessing the severity of insomnia symptoms (a 5-point response scale). The ISI demonstrated high internal consistency, ranging from *ω* = .88 to .92. Descriptive statistics for average scores by wave are presented in Supplementary Table 1E.

### Analysis

To estimate network models from the longitudinal data, we employed the GVAR model (Epskamp, 2020) using the *psychonetrics* (Epskamp, 2023) and *qgraph* (Epskamp, Cramer, Waldorp, Schmittmann, & Borsboom, 2012) packages. The analyses were conducted in R version 4.2.1 (R Core Team, 2023). The GVAR models were estimated separately for each of the four constructs (i.e. depression, anxiety, PTSD, and insomnia). To handle missing data, we used full information maximum likelihood (FIML) estimation. We pruned the networks at a significance threshold of .05. We quantified the centrality of the individual symptoms as the in- and out-strength for the temporal networks and as strength in the contemporaneous networks (in temporal networks: in-strength = the sum of the weights of all incoming edges, out-strength = the total weight of outgoing edges; in contemporaneous and between-person networks: strength = the sum of the absolute values of all edge weights connected to a node). For each network, we evaluated the stability of the edges using a bootstrapping procedure (100 iterations, drawing 75% of the full sample for each iteration; see Freichel, 2023). The code used for the analyses is available at https://osf.io/dfrqp/.

## Results

**Figure 1.**
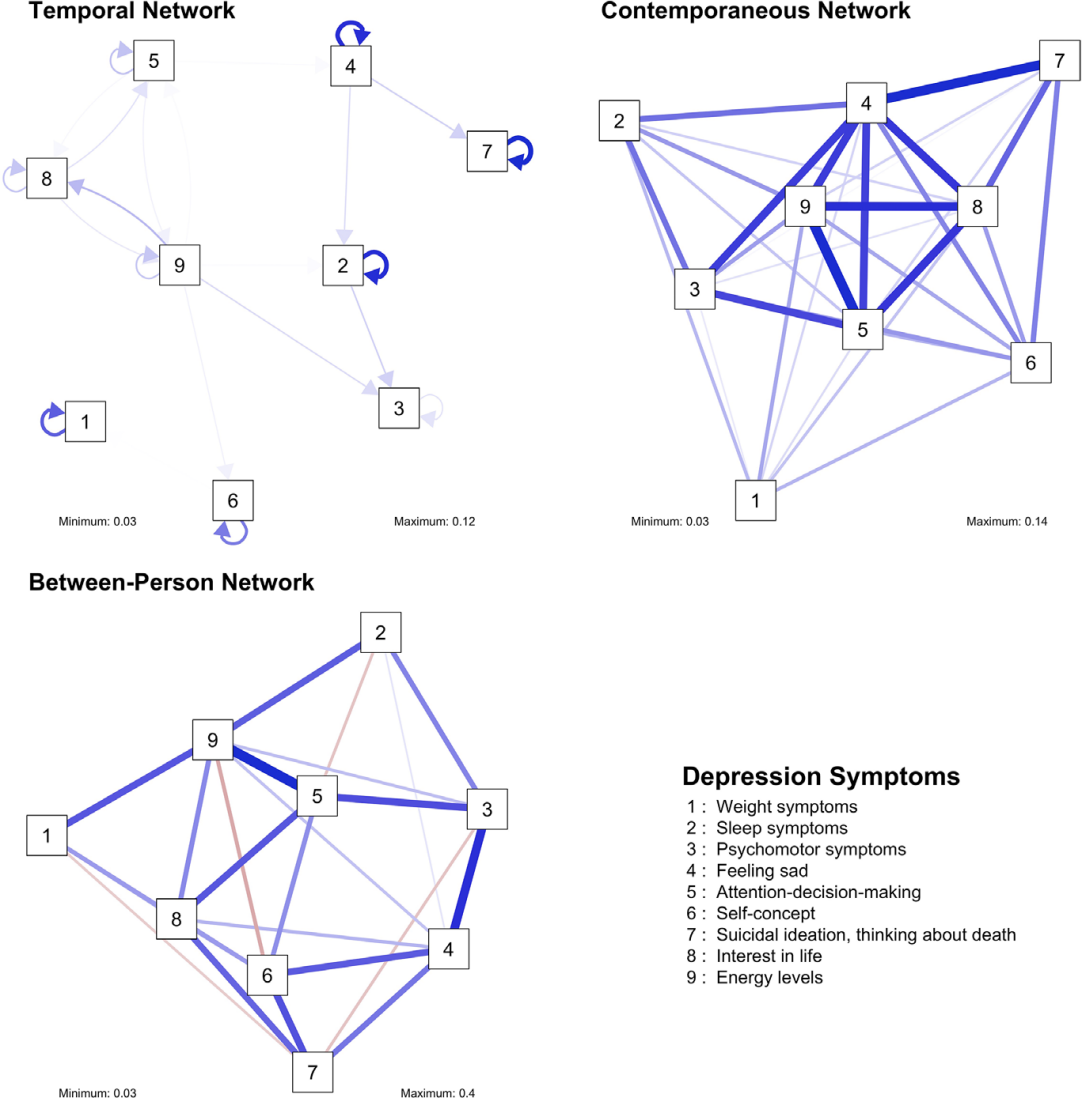
Visualizations of the depression networks.

**Figure 2.**
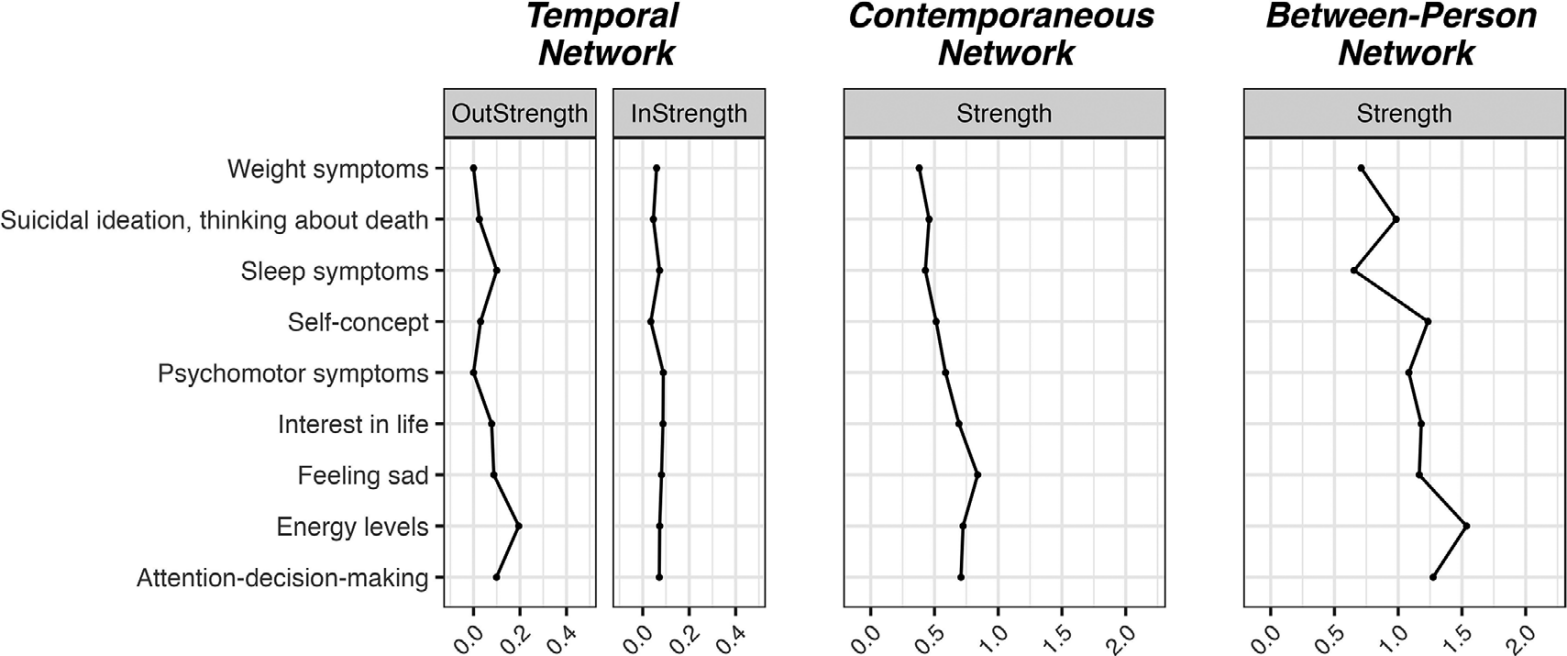
Centrality measures of the depression networks.

**Figure 3.**
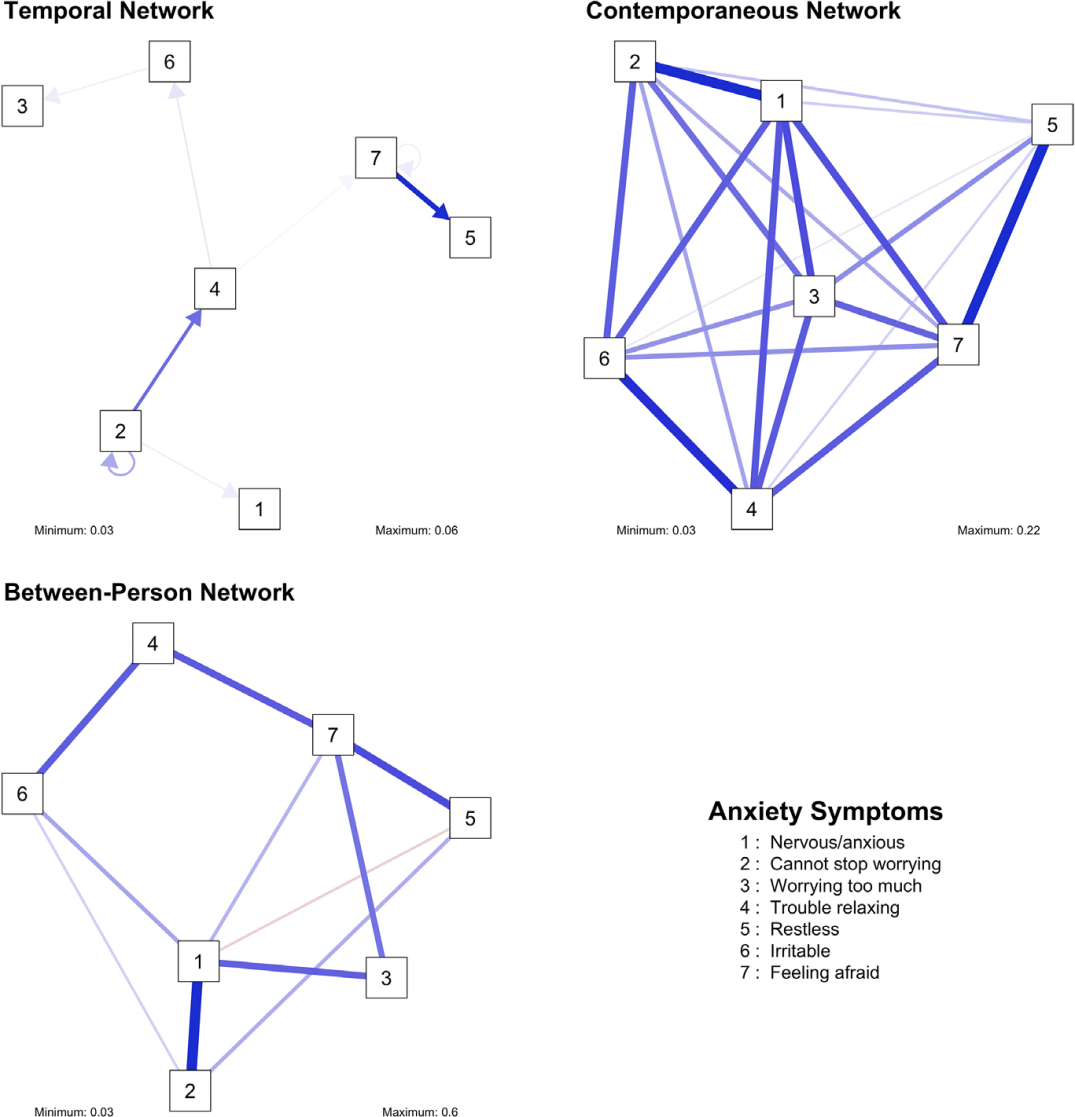
Visualizations of the anxiety networks.

**Figure 4.**
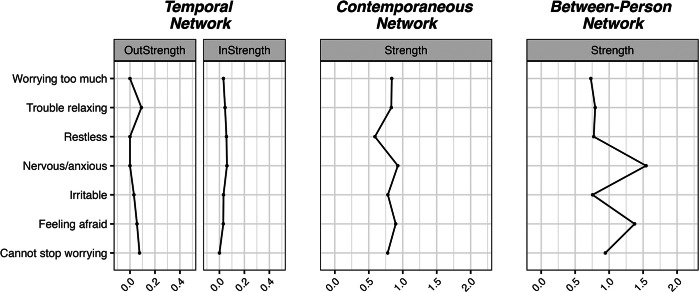
Centrality measures of the anxiety networks.

**Figure 5.**
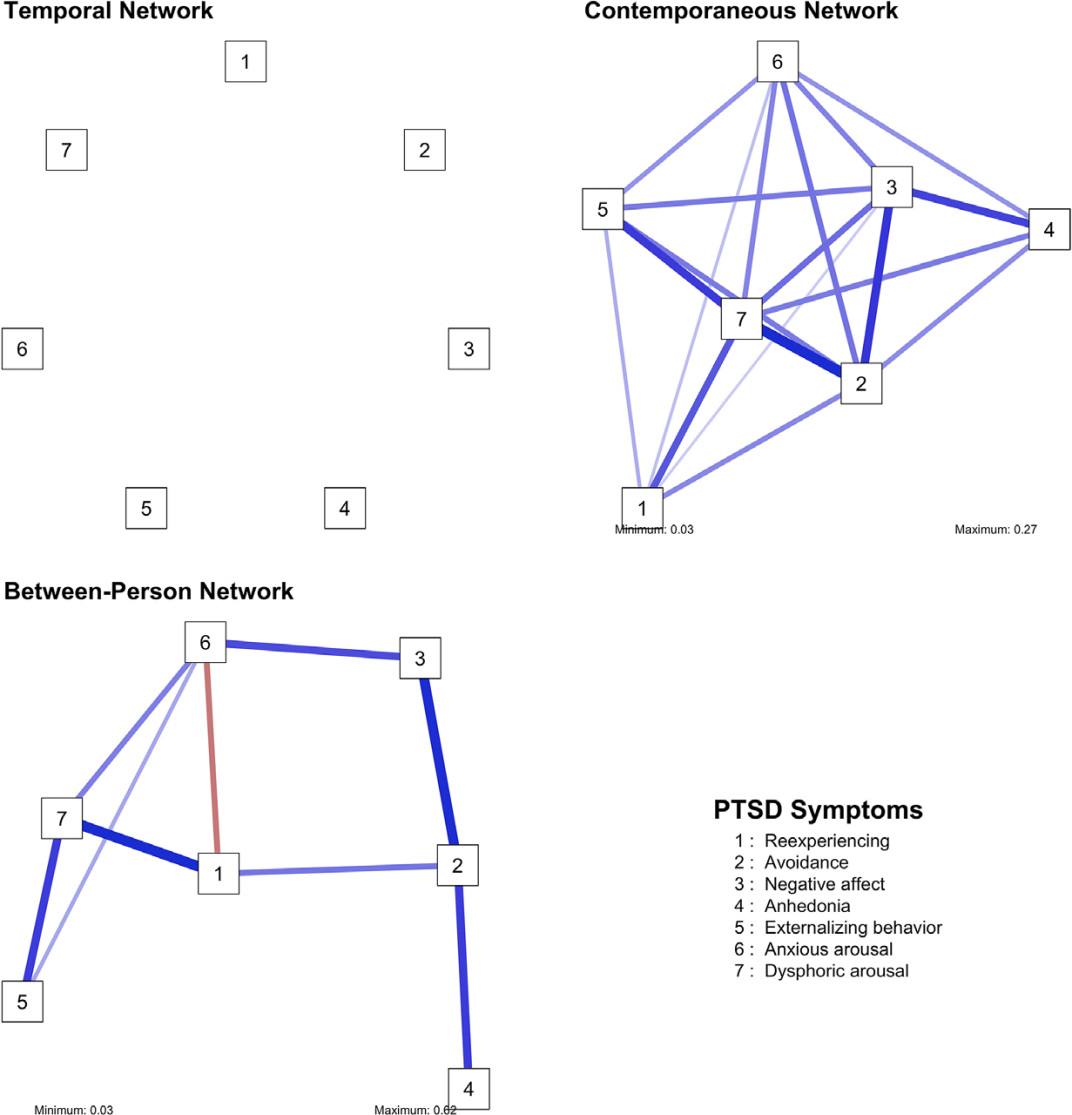
Visualizations of the PTSD networks.

**Figure 6.**
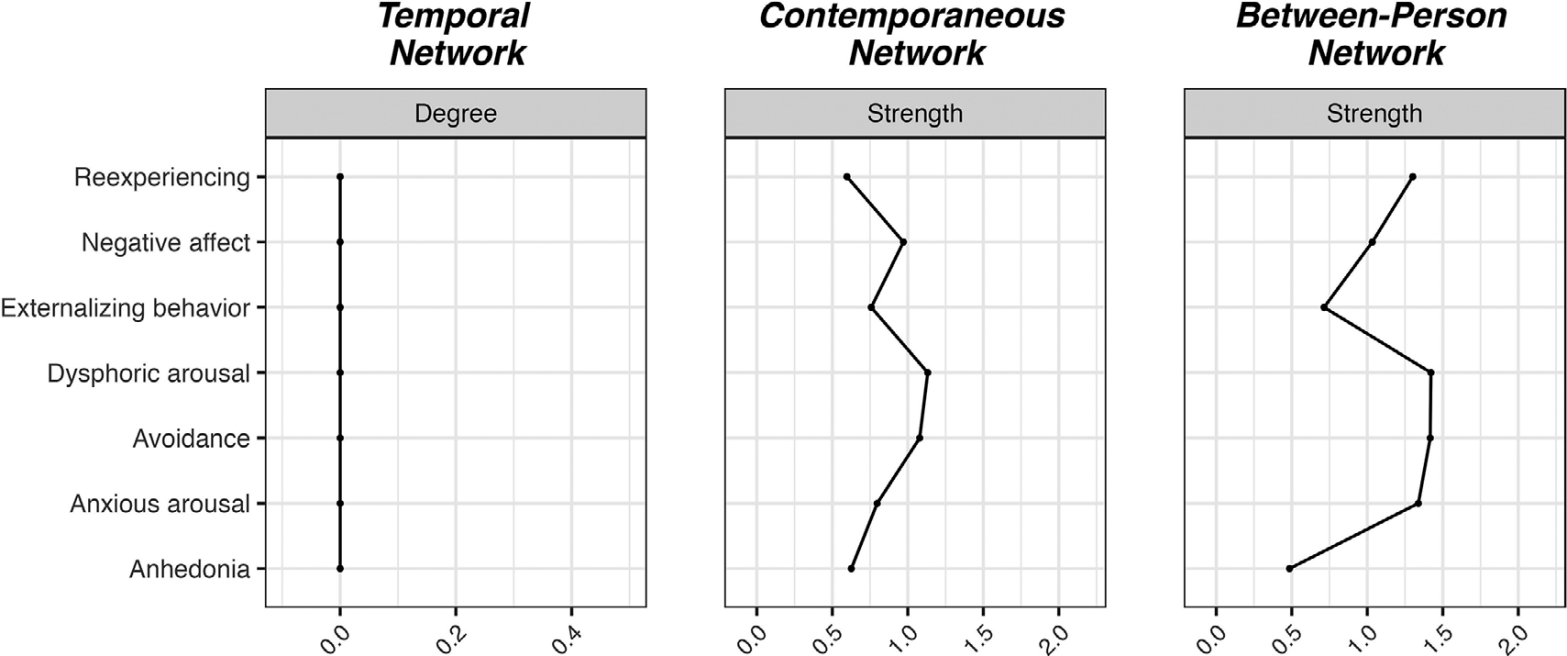
Centrality measures of the PTSD networks.

**Figure 7.**
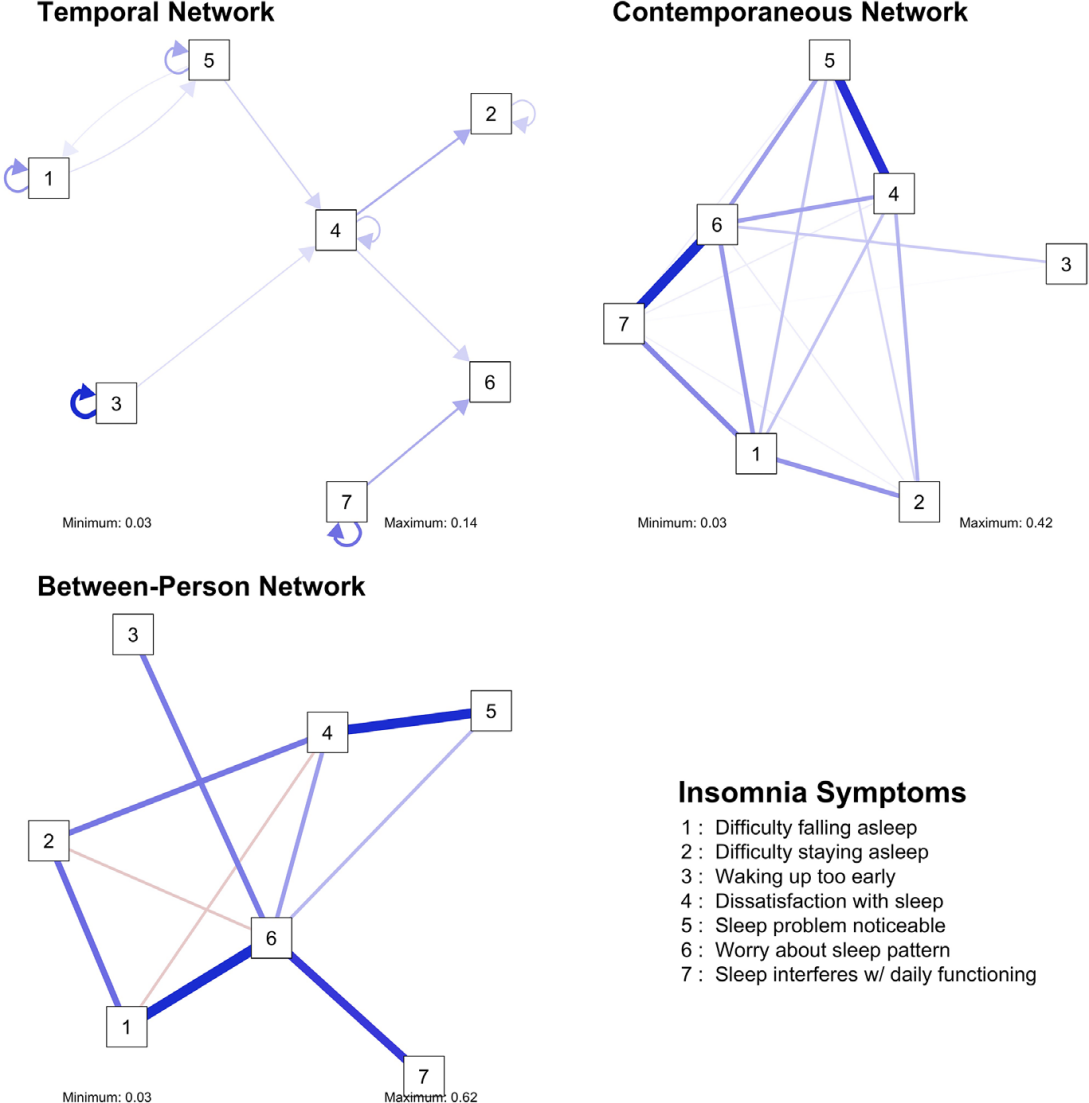
Visualizations of the insomnia networks.

**Figure 8.**
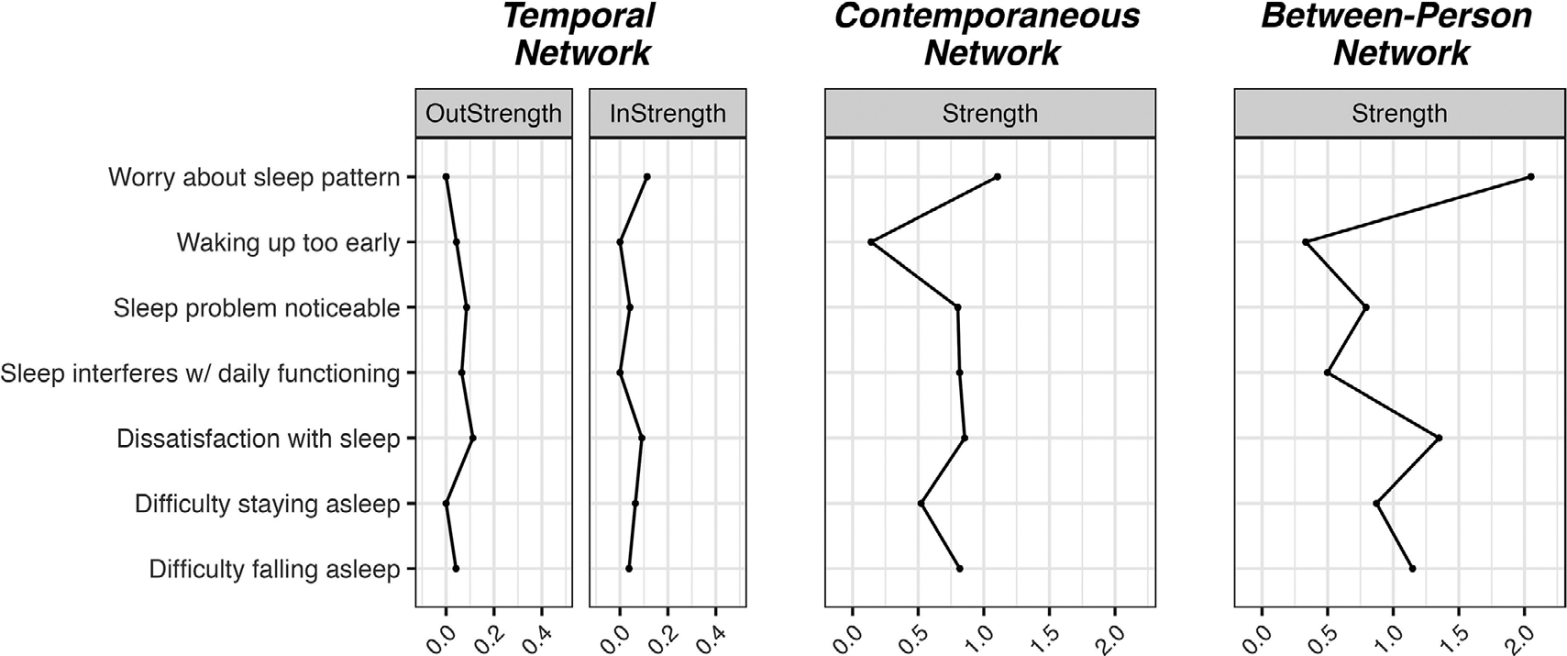
Centrality measures of the insomnia networks.

The main results are summarized below separately for each construct. Model fit indices for each network are reported in Table 1. The results of the sensitivity checks are available in a shiny app: https://benjamin-imsa.shinyapps.io/mh_slovakia_networks/. A full table of edge weights, as well as the outcomes of the bootstrapping analyses, are available in the Supplementary Material. Visualizations of the networks and their centrality indices are available in Figures 1–8. For clarity, we omitted edges with weights below 0.03 from all visualizations.

**Table 1.** Fit measures of the network models

	Depression	Anxiety	Insomnia	PTSD
CFI	.88	.86	.86	.97
TLI	.87	.86	.86	.97
RMSEA	.02	.04	.02	.03

*Note*: The depression network consists of nine symptoms measured by the QIDS-SR-16; the anxiety network consists of seven symptoms measured by the GAD-7; the PTSD network consists of seven symptom clusters derived from 20 PTSD symptoms measured by the PCL-5; and the insomnia network consists of seven symptoms measured by the ISI.

### Depression

We identified eight significant cross-lagged edges in the temporal network: sleep symptoms positively predicted psychomotor symptoms, feeling sad positively predicted sleep symptoms and suicidal thoughts at the next timepoint, while energy levels positively predicted psychomotor symptoms. There was a positive feedback loop between attention-decision-making and interest in life, as well as between interest in life and energy levels. All nodes in the temporal network had significant autoregressive effects (with sleep symptoms, feeling sad, and suicidal thoughts showing the strongest ones). However, the strength of the edges was relatively low in the temporal network (maximum: 0.12).

The contemporaneous network of depression symptoms was densely connected, with the associations between feeling sad and suicidal thoughts, and between energy levels and attention-decision-making being the strongest ones. On the between-person level, the network is dense, and the strongest association is the one between attention-decision-making and energy levels.

We also estimated the in- and out-strength as centrality measures for the temporal network and strength for the contemporaneous network. In the temporal network, energy levels and sleep symptoms emerged as two indicators with comparatively highest out-strength. Three symptoms had similarly high in-strength – psychomotor symptoms, interest in life, and feeling sad. Feeling sad was the most central symptom in the contemporaneous network.

### Anxiety

In the temporal network, the edge weights were relatively low (maximum = 0.1). Two notable patterns emerged in the network. The most salient is the chain of four variables: being unable to stop worrying positively predicts having trouble relaxing, which in turn positively predicts being irritable, which then predicts worrying too much. Feeling afraid is the only stable node, with a significant autoregressive effect. In the dense contemporaneous network, the strongest edges are between feeling nervous/anxious and worrying, and between feeling restless and feeling afraid. In the between-person network, only the positive association between having trouble relaxing and being irritable, and the positive association between being restless and feeling afraid were significant. Not being able to stop worrying was the anxiety symptom with the highest out-strength, while feeling restless had the highest in-strength. Feeling nervous/anxious and feeling afraid were the strongest nodes in the contemporaneous network.

### PTSD

We identified no significant edges in the temporal network of PTSD. This may be due to the fact that the interval between the PTSD measurements was twice as long as that of the other studied variables, suggesting that these symptoms interact on shorter time scales. On the other hand, the contemporaneous network was densely connected with the edges between avoidance and dysphoric arousal, and between negative affect and avoidance being the strongest ones. In the between-person network, the following edges emerged as the strongest ones: re-experiencing – dysphoric arousal, negative affect – avoidance, and externalizing behavior – dysphoric arousal. Dysphoric arousal and avoidance emerged as the most central symptoms in the contemporaneous network.

### Insomnia

In the rather dense temporal network, the two strongest edges were: (i) dissatisfaction with sleep positively predicted difficulties staying asleep and (ii) sleep interfering with daily functioning positively predicted worrying about sleep patterns. The autoregressive effects were the strongest for waking up too early and sleep interfering with daily functioning.

The two strongest edges in the contemporaneous network were the positive associations between worrying about sleep patterns and sleep interfering with daily functioning, and between dissatisfaction with sleep and noticeable sleep problems. In the between-person network, the same two associations were also the strongest. In the temporal network, waking up too early had the highest out-strength, while worrying about sleep patterns was the symptom with the highest in-strength. Worrying about sleep patterns was also the most central symptom in the contemporaneous network.

## Discussion

The present study examined the structure and temporal dynamics of four main mental health constructs—depression, anxiety, PTSD, and insomnia—using the network approach in a representative sample of the general population over a 3.5-year period. All four constructs exhibited relatively densely connected contemporaneous networks, suggesting relatively strong associations among symptoms within the same measurement window. However, the temporal networks showed notable differences. Depression and insomnia showed more (significant) cross-lagged effects compared to anxiety and PTSD. This variation in temporal dynamics might reflect differences in the stability and mutual influence of symptoms across disorders, with stronger temporal connections suggesting more predictable day-to-day symptom progressions, as opposed to more context-dependent symptom fluctuations in a weakly connected temporal networks (alternatively, sparse temporal networks may indicate that the symptom interactions occur on a timescale different from the one captured in the present study). The between-person networks revealed varying connectivity across disorders, highlighting stable, trait-like symptom associations at the population level. With the exception of insomnia, the observed symptom interaction patterns markedly differed from those observed in temporal and contemporaneous networks, underscoring the importance of distinguishing within-person processes from between-person differences in symptom structures.

We now turn to a more detailed discussion of each individual disorder, highlighting the potential implications of its network characteristics.

### Depression

The results underscore the need for a multifaceted understanding and holistic treatment of depression. The similar in-strength parameters across symptoms indicate only minor variability in the overall magnitude of how symptoms affect each other. In contrast, the notable variability in out-strength highlights candidates for potential interventions: decision-making, sleep problems, and low energy. The high interconnectedness of sadness with other symptoms highlights the importance of addressing core emotional disturbances in depression treatment (Marroquín & Nolen-Hoeksema, 2015). The centrality estimates further emphasize the interdependence of cognitive and motivational aspects of depression (Grahek, Shenhav, Musslick, Krebs, & Koster, 2019). Feelings of sadness and loss of interest in life are causes of two pathways involving low energy and sleep problems, which together lead to deteriorated psychomotor capabilities. Sleep problems, in particular, have been well-documented as a risk factor for depression (Zhang et al., 2022), highlighting the potential for targeted interventions in this area to mitigate broader symptoms of depression.

### Anxiety

The most notable insight into the dynamics of anxiety is the existence of two distinct temporal chains. In the first, feeling afraid predicts both restlessness and itself, indicating persistently heightened arousal and vigilance (see, e.g. Blakey & Abramowitz, 2016). In the second chain, an inability to stop worrying is a common cause of nervousness and trouble relaxing. The latter then exacerbates irritability and excessive worrying. This suggests a feedback loop where cognitive symptoms (worrying) intensify physical symptoms (trouble relaxing and irritability), which in turn reinforce cognitive distress. This highlights the cyclical nature of anxiety symptoms and provides evidence for the cognitive model of anxiety (Wells, 1999). The highly dense contemporaneous network suggests that anxiety symptoms frequently co-occur and intensify each other simultaneously, implying that interventions targeting one symptom may alleviate others.

### PTSD

The complete lack of significant temporal connections suggests that the persistence of PTSD symptoms may be driven more by individual’s environment and situational factors than by symptom progression and interactions over time. This highlights the importance of interventions that are responsive to the person’s immediate context and emotional state (e.g. Timulak & Keogh, 2020). The contemporaneous network indicates that individuals who avoid trauma reminders tend to experience elevated dysphoric arousal and are more likely to feel heightened distress. Additionally, negative affect strongly drives avoidance behaviors, suggesting that emotional distress contributes to disengagement from trauma-related stimuli. The high centrality of avoidance and dysphoric arousal highlights their critical role in PTSD symptomatology. Interventions targeting these symptoms, such as those aimed at reducing avoidance behaviors or managing dysphoric arousal, could have a cascading effect in alleviating other PTSD symptoms. This aligns with evidence supporting the efficacy of emotion regulation strategies in PTSD treatment (Kaczkurkin et al., 2017), which may also help reduce associated behaviors such as externalizing tendencies, including reckless or risky behavior (Weiss, Tull, & Gratz, 2014). In the between-person network, strong associations were identified between re-experiencing and dysphoric arousal, between negative affect and avoidance, and between externalizing behavior and dysphoric arousal. These findings suggest that dysphoric arousal serves as a bridge between re-experiencing trauma-related memories and externalizing behaviors, such as impulsivity or aggression. Negative affect and avoidance also appear closely linked, highlighting how emotional distress can drive avoidance behaviors, which in turn may hinder recovery by preventing exposure to trauma-related stimuli necessary for processing and habituation (e.g. McLean, Levy, Miller, & Tolin, 2022).

### Insomnia

The temporal network suggests a mechanism where the impact of sleep issues on daily life increases anxiety about sleep, further exacerbating sleep problems and aligning with cognitive models of insomnia (Tang, Saconi, Jansson-Fröjmark, Ong, & Carney, 2023). Notably, strong autoregressive effects for waking up too early and sleep interfering with daily functioning highlight the persistence of these issues over time. In the contemporaneous network, the strongest associations were between worrying about sleep patterns and sleep interfering with daily functioning, and between dissatisfaction with sleep and perceived sleep problems. Waking up too early had the highest out-strength, indicating its strong influence on other symptoms, while worrying about sleep patterns showed the highest in-strength and was the most central symptom in the contemporaneous network. This centrality implies that behavioral interventions targeting sleep duration to prevent early awakening and addressing sleep-related worries (e.g. CBT-I; Trauer, Qian, Doyle, Rajaratnam, & Cunnington, 2015) could help mitigate multiple symptoms of insomnia.

### Limitations and future directions

There are several limitations to this study. First, due to the longitudinal design, the representativeness of the sample slightly diminished over time, with the mean correlation with population parameters, based on Slovak Statistical Office data (2021), at .75. Given that the effects of mental health scores on dropout status were minimal (average Hedges’ *g*s ranging from 0.00 to 0.13), it is likely that other factors contribute more substantially to attrition. However, the specific reasons for dropout remain unknown. Additionally, sample attrition over the 3.5-year period reduces the power to detect smaller lagged effects to the extent that participants without consecutive-wave data cannot inform lag-1 temporal effects, even when using FIML. Third, there are several limitations to our modeling approach. Our models assumed relationships to be linear. Modeling only lag-1 effects might overlook delayed effects not captured by lag-1 relations. GVAR models also assume stationarity, which may be violated in the presence of time trends. Subsequent sensitivity analysis with detrending, however, showed no substantial alterations in the results. Additionally, our conclusions depend on the chosen measurement intervals, potentially omitting important variability at alternative time scales. Finally, while some existing findings are promising in suggesting that interventions based on network analysis (e.g. centrality analysis) may be effective (Castro, Gysi, Ferreira, Ferreira-Santos, & Ferreira, 2024; Rodebaugh et al., 2018), several challenges remain to be addressed (e.g. Bringmann et al., 2019). Consequently, the premise that network models can reliably guide treatment selection is still subject to empirical validation (e.g. Lunansky et al., 2022; Ryan & Hamaker, 2022).

Overall, this study provides novel insights into the dynamics of mental health constructs in a representative sample of the general population tracked over 3.5 years (a period of major societal disruptions, including COVID-19 and the war in neighboring Ukraine) and provides for a more nuanced understanding of how mental health symptoms interact over time and across individuals. The findings can assist practitioners and public health experts in better understanding the complex nature of these four mental health indicators, which could be instrumental in developing targeted interventions, particularly for addressing persistent mental health conditions. Future studies should explore broader life circumstances to uncover heterogeneity in mental health dynamics. A deeper understanding of these dynamics can aid in the development of preventive strategies, ultimately contributing to better public health outcomes.

## Data Availability

All data, R scripts, and other Supplementary Material are available at https://osf.io/dfrqp/.
